# Structured correction after difficult direct laryngoscopy during prehospital rapid sequence intubation: an eleven-year physician-staffed helicopter emergency medical service registry study

**DOI:** 10.1186/s12245-026-01342-7

**Published:** 2026-08-11

**Authors:** Akos Soti, Peter Temesvari, Laszlo Hetzman, Robert Gebei, Bank G. Fenyves, Csaba Varga

**Affiliations:** 1 Department of Emergency Medicine, Semmelweis University, Budapest, Hungary; 2 Hungarian Air Ambulance Nonprofit Ltd., Budaörs, Hungary; 3 Faculty of Health Sciences, Institute of Emergency, Health Pedagogy and Nursing Sciences, University of Pécs, Pécs, Hungary; 4 Department of Emergency Medicine, Kaposi Mór Teaching Hospital, Kaposvár, Hungary; 5 Emergency Medical Centre, North-Pest Central Hospital-Military Hospital, Budapest, Hungary

**Keywords:** Intubation, Airway management, Emergency medical service, Laryngoscopy, Rapid sequence intubation, 30-second drill

## Abstract

**Background:**

Difficult laryngoscopy during prehospital rapid sequence intubation may lead to procedural failure and serious adverse outcomes. Evidence is limited regarding whether a structured correction bundle can convert an initial poor view into successful intubation before declaring failure. We evaluated the outcomes of a structured correction protocol used after a poor initial laryngoscopy view during physician-staffed helicopter emergency medical service rapid sequence intubation.

**Methods:**

We conducted a retrospective observational study using prospectively collected data from the Hungarian Air Ambulance airway registry. Patients older than 14 years with maintained circulation who underwent a tracheal intubation attempt between 2012 and 2022 were included. The primary endpoint was the proportion of successful tracheal intubations after at least one corrective maneuver among cases requiring corrective maneuvers. Secondary endpoints included the success rates of individual maneuvers and associations between corrective maneuvers and prespecified covariates.

**Results:**

Among 2,431 included patients, 1,827 (75.2%) intubations were successfully performed without the need for any corrective maneuvers. The primary endpoint, frequency of successful intubations after at least one corrective maneuver, was achieved in 578 out of 604 cases (95.7%; 95% CI 98.4–99.3). First-pass success in the full cohort was 92.8% (2,255/2,431; 95% CI 91.7–93.8), and overall intubation success was 98.9% (2,405/2,431; 95% CI 98.4–99.3). In 63.7% (385 of 604) of the correction cases, only one maneuver was required. The most common single maneuvers were suction and backward-upward-rightward pressure, each associated with 100% success. The combination of multiple correction elements was also associated with a high success rate. Male sex, cricoid pressure, head-elevated laryngoscopy position, and non-anesthesiology operator specialty were all associated with a higher risk of requiring corrective maneuvers.

**Conclusion:**

In this standardized prehospital rapid sequence intubation system, a structured correction protocol after an initially poor direct laryngoscopy view was associated with high rates of subsequent intubation success.

**Supplementary Information:**

The online version contains supplementary material available at 10.1186/s12245-026-01342-7.

## Background

Endotracheal intubation is considered the gold standard for advanced airway management in critically ill patients in the prehospital setting. Its clinical benefit, however, depends strongly on provider training, system design, patient selection, and procedural quality [1, 2]. Performing the procedure without complications and having a detailed plan for anticipated failure are therefore central to safe practice [3, 4]. Recent international guidelines describe steps after failed laryngoscopy (a rare but potentially fatal event) but offer limited evidence on corrective maneuvers used during tracheal intubation attempts [4–14].

In 2011, Hungarian Air Ambulance Nonprofit Ltd. (HAA) introduced a standard operating procedure (SOP) for rapid sequence intubation (RSI), informed by the London’s Air Ambulance model [15–18]. This protocol defines a structured set of corrective steps to be used after an unsuccessful laryngoscopy, allowing potentially modifiable factors to be addressed before the procedure is classified as a failure. Although several individual corrective maneuvers have been evaluated previously, the effectiveness of the full correction package has not been studied. The introduction of the protocol was accompanied by the implementation of a mandatory airway registry, with data recorded in accordance with international standards [19].

HAA is a government-owned and financed service operating seven daytime helicopter bases nationwide. It is the country’s only helicopter emergency medical service (HEMS) and is dispatched by National Ambulance Service control centers. Standard crew configuration consists of a physician, a paramedic, and a pilot. Regular medical training, simulation, and post-mission debriefing are integral to operations. The attending physician is an anesthesiologist or an emergency physician, occasionally from another specialty, and may be a specialist or a specialty trainee (with at least two years of training). The service performs approximately 250 rapid sequence intubations per year. No separate national difficult-airway algorithm exists in Hungary. The national ground-based ambulance service’s RSI SOP is itself based on the HAA protocol.

## Methods

The primary aim of this study was to evaluate the outcomes of a structured correction protocol in cases in which corrective maneuvers were used during prehospital rapid sequence intubation. A secondary aim was to explore associations between demographic characteristics, treatment-related factors, and both the need for and outcome of corrective maneuvers.

### Study design, setting, and participants

We conducted a retrospective observational study using prospectively collected HAA airway registry data on prehospital rapid sequence intubations performed between January 1, 2012, and December 31, 2022 (eleven calendar years, inclusive). Patients older than 14 years with maintained circulation who underwent at least one tracheal intubation attempt by the HEMS crew were included. This study is reported in accordance with the Strengthening the Reporting of Observational Studies in Epidemiology (STROBE) statement [20].

### Airway protocol

The rapid sequence intubation protocol is consistent with international guidelines [5, 8–13]. Equipment is standardized and checked daily, and preparation includes a standard equipment layout, preoxygenation and apneic oxygenation, optimization of patient and operator positions, hemodynamic stabilization when needed, and pre-drawn induction and neuromuscular blocking drugs. The operator and assistant complete a challenge-response checklist to optimize the first attempt and prepare rescue steps, and a gum elastic bougie is used routinely. The full standard operating procedure and checklist are provided in the Supplementary material.

**Fig. 1:**
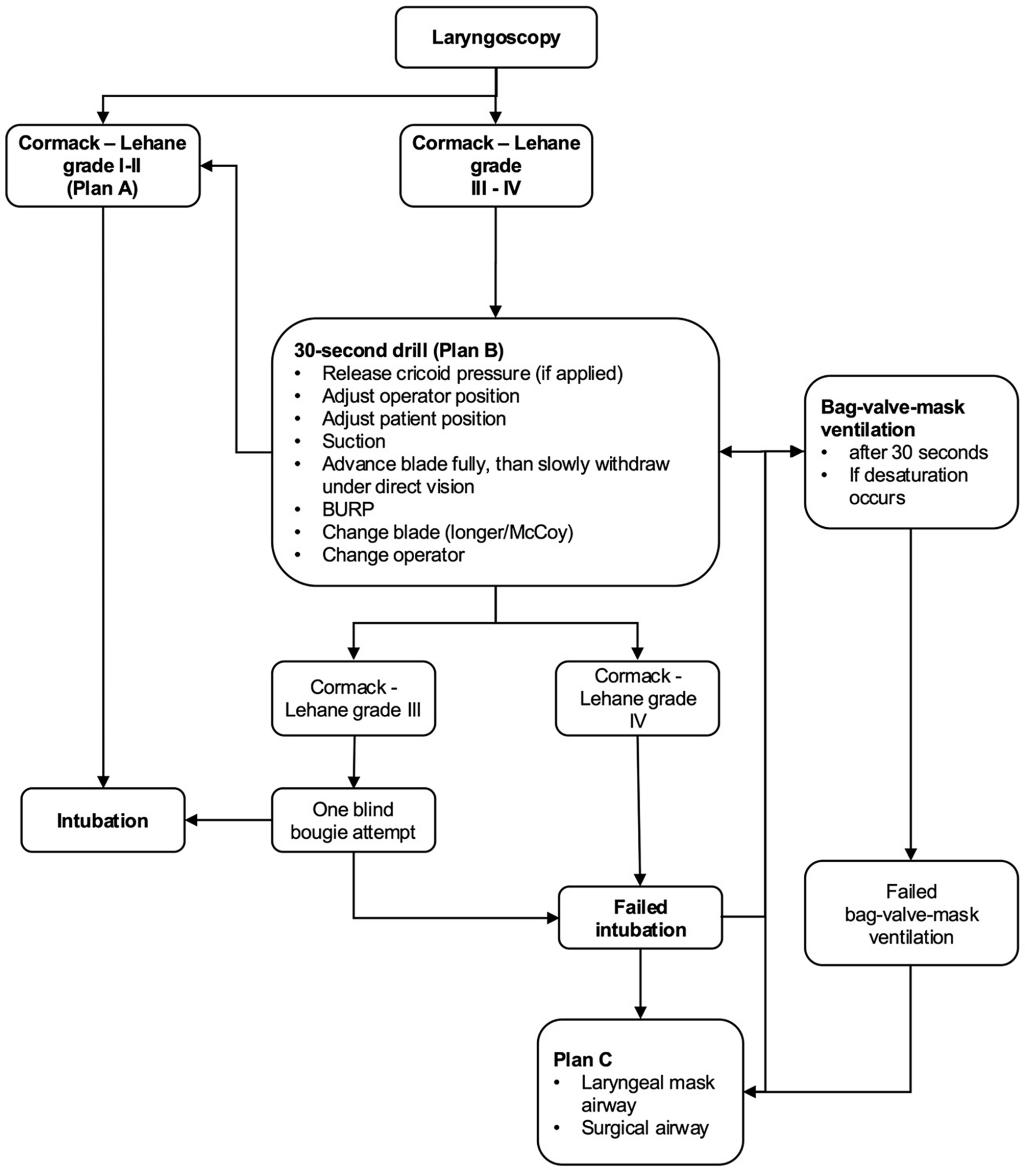
Intubation flowchart

Plan A is the initial direct-laryngoscopy attempt, aiming for a Cormack-Lehane grade I or II view and first-pass success. If the view is grade III or IV, Plan B (the “30-second drill”) is initiated, a sequence of corrective actions to improve the view during laryngoscopy, summarized in Table 1 [5, 9]. The team has up to 30 s before ventilating the patient, and the drill is interrupted for bag-valve-mask ventilation if desaturation occurs. Within no more than two laryngoscopy attempts, one blind bougie attempt is accepted in a grade III view. Otherwise, failed laryngoscopy is declared, and the team proceeds to Plan C, laryngeal mask airway insertion or surgical airway access (Fig. 1).

**Table 1:** Elements of the 30-second drill for difficult laryngoscopy

Corrective maneuver	Practical trigger/rationale	Practical application in the SOP	Important caveats / additional details
Release cricoid pressure	Cricoid pressure (also known as the Sellick maneuver) is pressure applied to the cricoid cartilage to occlude the esophagus and prevent regurgitation during laryngoscopy [21]. It was historically part of the RSI procedure. Its role has been debated. It does not reliably reduce the risk of aspiration and may increase the difficulty of intubation [22].	First corrective step when cricoid pressure is present.	It was initially part of RSI for obese, pregnant, and bag-valve-mask ventilated patients, but in recent years it has been removed from the SOP.
Adjust operator position	The operator’s position is a key element of success. The operator must be on the same axis as the patient and in a stable position [9, 23].	Check whether the operator is standing, kneeling on both knees, or lying prone as appropriate for patient height, correct instability, axis deviation, or height deviation.	Position should already be optimized before preparation and double-checked during the challenge-response checklist. During difficult laryngoscopy, the assistant should check and verbalize whether the difficulty may be due to operator instability or axis/height deviation.
Adjust patient position	Suboptimal patient positioning may impair glottic visualization [24–29].	Patient position is reassessed and corrected. 360-degree access and appropriate shade are essential. Sniffing as standard position, occipital padding + Manual In-Line Stabilization (MILS) for trauma patients, Head-Elevated Laryngoscopy Position (HELP) for the obese	Position should already be optimized before preparation and double-checked during the challenge-response checklist. During difficult laryngoscopy, the assistant should check and verbalize whether the difficulty may be due to inappropriate patient positioning.
Suction	Blood, saliva, or regurgitated material may obstruct the laryngoscopy view [30].	The airway is suctioned immediately with a rigid, large-bore tube, when contamination impairs visualization.	If the view is not obstructed and the glottis can be visualized, intubation should proceed. Any solid aspiration can be removed with Magill forceps.
Advance blade fully, then slowly withdraw under direct vision	The blade may be too deep or off axis, resulting in loss of glottic view or disorientation.	The blade is advanced to maximal depth and then slowly withdrawn under direct vision to identify the glottis.	This maneuver may bring the glottis into view [31].
Backward-upward-rightward pressure (BURP)	External laryngeal manipulation may improve laryngeal view [5, 32–34].	The operator guides backward-upward-rightward pressure while maintaining visualization; the assistant maintains the optimal position once view improves.	It is often confused with Sellick’s maneuver, although the two interventions have completely different aims. The operator adjusts a second assistant’s hand to the correct position while maintaining the laryngoscopy view. The first assistant’s hands must remain free of this maneuver, as their actions will be required during intubation.
Change blade (longer or McCoy)	Inappropriate blade size or blade type may limit laryngoscopy.	The blade is changed when size selection appears suboptimal or when a McCoy blade is judged likely to improve epiglottic elevation.	A blade that is too large is usually not a problem, but one that is too small can cause difficulties. A spare laryngoscope can be used in the event of sudden failure (laryngoscopes are checked during the checklist). A large epiglottis can be addressed with McCoy-type blades [35].
Change operator	Rarely, the initial operator may be unable to continue because of internal or external factors.	If another trained operator is available, the operator role may be transferred [5].	This might also happen during trainee supervision.

Abbreviations: BURP, backward-upward-rightward pressure; RSI, rapid sequence intubation; SOP, standard operating procedure

The drill is a team procedure, with the assistant monitoring time and oxygen saturation and considering corrective steps in sequence unless a clearly indicated intervention, such as suction, is needed immediately. Not all maneuvers are used in every case, but all are considered. The decision to interrupt the drill for bag-valve-mask ventilation is a clinical judgment rather than a fixed SpO₂ threshold, integrating the patient’s position on the oxyhemoglobin dissociation curve, the known delay of pulse oximetry relative to arterial oxygenation, and the patient’s baseline oxygenation.

### Data source and variables

Data were extracted in anonymized form from the electronic patient record system. Registry data were entered by the treating physician immediately after the mission as part of routine clinical documentation. Recorded variables included demographics, initial and best Cormack-Lehane grade, number of laryngoscopy attempts, intubation success, airway optimization maneuvers used individually and in combination, and rescue actions undertaken after failed intubation. Prespecified covariates were sex, age, patient category defined by primary underlying pathology, patient position during intubation, crash intubation, use of cricoid pressure, and operator specialty (emergency medicine, anesthesiology and intensive therapy, or other) and grade (specialist or in training). Crash intubation was defined as an accelerated induction sequence performed when the patient’s condition did not allow time for standard preparation, performed with an abbreviated checklist and without full optimization. A neuromuscular blocking agent was always administered, whereas an induction agent was given selectively according to circulatory status and level of consciousness.

Missing values are reported in the corresponding tables, and cases with missing values were excluded from analyses involving those variables. Analyses were conducted on complete cases. The proportion of missing data for each covariate is reported in Table 2 (patient position 11.7%, patient category 8.1%, sex 2.3%, and operator specialty and grade 0.4% each, with age, crash intubation, and cricoid pressure fully recorded), and 79.4% of patients had complete data for all covariates. No imputation was performed.

### Outcomes

The primary endpoint was the frequency of successful tracheal intubation after application of at least one corrective maneuver, assessed in all cases in which one or more corrective maneuvers were used. Successful tracheal intubation was defined as correct tube placement confirmed by capnography. Although the correction protocol is triggered by a Cormack-Lehane grade III or IV view, we defined the analyzed cohort by the documented use of at least one corrective maneuver rather than by the recorded initial Cormack-Lehane grade.

Secondary endpoints were the success rates of individual corrective maneuvers and their combinations, and the associations between corrective maneuvers and demographic characteristics, prespecified covariates, and treatment-related factors. First-pass success was defined as successful tracheal intubation achieved during the first laryngoscopy, without a further laryngoscopy attempt, including cases in which corrective maneuvers were applied during that first laryngoscopy with the laryngoscope blade kept in place.

### Statistical analysis

Logistic regression models were used to evaluate associations between prespecified covariates and the use and outcomes of corrective maneuvers. In cases in which only one corrective maneuver was used, multinomial modeling was performed. Because low frequencies of many specific corrective-maneuver combinations precluded stable multinomial modeling, targeted binary logistic regression models were fitted for more common combinations. Results were reported as odds ratios and relative risk ratios with 95% confidence intervals. 95% confidence intervals for success proportions were calculated using the Clopper-Pearson exact method. Statistical significance was defined as a two-sided p-value < 0.05. Reference categories for categorical predictors were prespecified and are reported in the corresponding tables. All prespecified covariates were entered simultaneously in a single multivariable model, without stepwise variable selection. For the primary model of any corrective-maneuver use, the 491 events among the 1,931 complete cases correspond to approximately 27 events per estimated parameter, adequate for stable estimation. Because several maneuver-specific and combination models were based on small numbers of events, these analyses were considered exploratory and hypothesis-generating. No adjustment for multiple comparisons was applied, and these estimates should be interpreted with caution given the potential for sparse-data bias and unstable estimation.

Data management was performed in Microsoft Excel for Microsoft 365 (Microsoft Corp, Redmond, WA), and statistical analyses in R version 4.5.2 (R Foundation for Statistical Computing, Vienna, Austria).

### Ethics

The study was approved by the Regional and Institutional Committee of Science and Research Ethics, South Buda Central Hospital–Szent Imre University Teaching Hospital (approval No. 5/2023; February 2, 2023).

During the preparation of this manuscript, the authors used ChatGPT (OpenAI) and Grammarly (Grammarly Inc.) solely to improve the language and readability of author-written text. The authors carefully reviewed and edited the resulting text and take full responsibility for the final content of the manuscript.

## Results

During the study period, Hungarian Air Ambulance completed 32,252 missions. Of these, 2,640 (8.2%) patients with maintained circulation required advanced airway management with tracheal intubation, and 2,431 (7.5%) met the inclusion criteria.

**Fig. 2:**
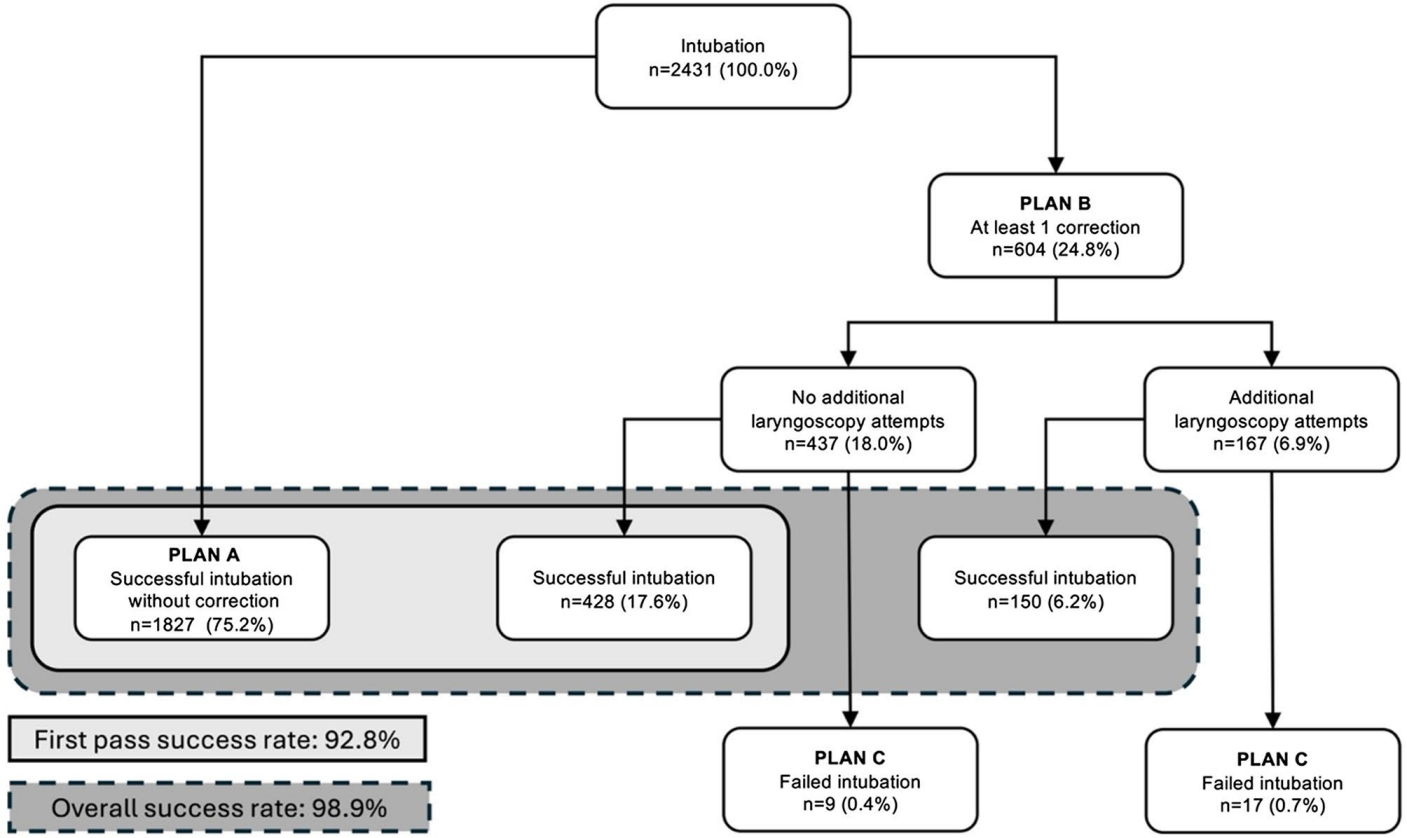
Distribution of intubation attempts and associated success rates

Among included patients, intubation was successful without use of corrective maneuvers in 1,827 cases (75.2%). In 604 cases, at least one corrective maneuver was used. In 437 of these, no additional laryngoscopy attempt was made, and intubation was successful in 428. Accordingly, first-pass success was achieved in 2,255 of 2,431 cases (92.8%; 95% CI 91.7–93.8). An additional 150 patients were successfully intubated after more than one laryngoscopy attempt, yielding an overall intubation success rate of 98.9% (2,405/2,431; 95% CI 98.4–99.3). Intubation failed in 26 patients (1.1%) (Fig. 2).

Patient and procedural characteristics are shown in Table 2.

**Table 2:** Patient and procedural characteristics

Variable	Category	No. (%)
Sex	Male	1641 (67.5)
	Female	734 (30.2)
	Missing	56 (2.3)
Age group, y	15–30	311 (12.8)
	31–60	1045 (43.0)
	61–85	951 (39.1)
	≥ 85	124 (5.1)
Patient category	Trauma	1115 (45.9)
	Neurological	437 (18.0)
	Internal medicine	326 (13.4)
	Intoxication	133 (5.5)
	Burns	120 (4.9)
	Asphyxia	40 (1.6)
	Other	64 (2.6)
	Missing	196 (8.1)
Patient position	MILS	1140 (46.9)
	Sniffing	799 (32.9)
	HELP	171 (7.0)
	Half-sitting	37 (1.5)
	Missing	284 (11.7)
Crash intubation	Yes	164 (6.7)
	No	2267 (93.3)
Cricoid pressure applied	Yes	63 (2.6)
	No	2368 (97.4)
Operator specialty	Emergency medicine	1206 (49.6)
	Anesthesiology and intensive therapy	769 (31.6)
	Other	447 (18.4)
	Missing	9 (0.4)
Operator grade	Specialist	1835 (75.5)
	In training	587 (24.1)
	Missing	9 (0.4)

Abbreviations: HELP, head-elevated laryngoscopy position; MILS, manual in-line stabilization

Percentages may not total 100 because of rounding

### Primary endpoint

The primary endpoint, frequency of successful intubations after at least one corrective maneuver, was achieved in 578 out of 604 cases (95.7%; 95% CI 98.4–99.3).

### Use of corrective maneuvers

The frequencies of individual corrective maneuvers are shown in Table 3. In 385 of 604 cases (63.7%), only one corrective maneuver was used, whereas in 219 cases (36.3%), more than one maneuver was used.

**Table 3:** Frequency of corrective maneuvers

Corrective maneuver	Correction cases, No. (%) (*n* = 604)	All included cases, No. (%) (*N* = 2431)	Used as sole maneuver, No.
Suction	355 (58.8)	355 (14.6)	197
BURP	294 (48.7)	294 (12.1)	123
Insert blade fully and withdraw under direct vision	112 (18.5)	112 (4.6)	14
Change blade (longer or McCoy)	107 (17.7)	107 (4.4)	25
Adjust patient position	49 (8.1)	49 (2.0)	9
Change operator	47 (7.8)	47 (1.9)	15
Adjust operator position	29 (4.8)	29 (1.2)	1
Release cricoid pressure	14 (2.3)	14 (0.6)	1

Abbreviation: BURP, backward-upward-rightward pressure

Percentages in the second column are based on 604 cases in which at least one corrective maneuver was used. Percentages in the third column are based on all 2431 included cases. More than one maneuver could be used in a single case. Therefore, counts are not mutually exclusive

The ten most frequently used corrective maneuvers or combinations of maneuvers, along with their associated success rates, are shown in Table 4. The most common single maneuvers were suction (*n* = 197) and BURP (*n* = 123), each with a 100.0% success rate.

**Table 4:** Most Frequently used corrective maneuvers and maneuver combinations, with associated success rates

Corrective maneuver or combination	No.	Success rate, % (95% CI)
Suction	197	100.0 (98.1–100.0)
BURP	123	100.0 (97.0-100.0)
Suction + BURP	46	97.8 (88.5–99.9)
Change blade (longer or McCoy)	25	100.0 (86.3–100.0)
BURP + insert blade fully and withdraw under direct vision	18	94.4 (72.7–99.9)
Change operator	15	100.0 (78.2–100.0)
Insert blade fully and withdraw under direct vision	14	92.9 (66.1–99.8)
Suction + change blade (longer or McCoy)	14	92.9 (66.1–99.8)
BURP + suction + change blade (longer or McCoy)	14	92.9 (66.1–99.8)
BURP + suction + change blade (longer or McCoy) + insert blade fully and withdraw under direct vision	13	69.2 (38.6–90.9)

Abbreviation: BURP, backward-upward-rightward pressure

### Factors associated with use of any corrective maneuver

In a multivariable binary logistic regression model including all prespecified covariates and operator specialty and grade (*n* = 1,931), male sex was associated with higher odds of requiring a corrective maneuver than female sex (OR 1.50; 95% CI 1.17–1.92). Use of cricoid pressure was also associated with greater odds of requiring a corrective maneuver (OR 3.33; 95% CI 1.76–6.27). Compared with the sniffing position, the HELP position was associated with greater odds of requiring a corrective maneuver (OR 2.74; 95% CI 1.88–4.01).

Compared with patients aged 31 to 60 years, patients aged 15 to 30 years had lower odds of requiring a corrective maneuver (OR 0.67; 95% CI 0.47–0.94). Internal medicine cases were less likely to require a corrective maneuver than trauma cases (OR 0.64; 95% CI 0.43–0.95). Compared with anesthesiology and intensive therapy, operators from emergency medicine (OR 1.48; 95% CI 1.15–1.90) and other specialties (OR 1.39; 95% CI 1.01–1.92) had higher odds of requiring a corrective maneuver, whereas operator grade (in training versus specialist) was not associated with the need for correction (OR 1.12; 95% CI 0.88–1.42) (Table 5).

**Table 5:** Factors associated with use of any corrective maneuver

Predictor	Reference category	OR (95% CI)	*P* value
Male sex	Female	1.50 (1.17–1.92)	0.001
Age 15–30 y	31–60 y	0.67 (0.47–0.94)	0.021
Internal medicine	Trauma	0.64 (0.43–0.95)	0.026
Cricoid pressure applied	No	3.33 (1.76–6.27)	< 0.001
HELP position	Sniffing	2.74 (1.88–4.01)	< 0.001
Operator: emergency medicine	Anaesthesiology and intensive therapy	1.48 (1.15–1.90)	0.002
Operator: other specialty	Anaesthesiology and intensive therapy	1.39 (1.01–1.92)	0.041
Operator in training	Specialist	1.12 (0.88–1.42)	0.37

Abbreviations: CI, confidence interval; HELP, head-elevated laryngoscopy position; OR, odds ratio

Results are from a multivariable binary logistic regression model including all prespecified covariates, operator specialty, and operator grade (*n* = 1,931)

### Factors associated with specific corrective maneuvers

Separate multivariable binary logistic regression models were constructed for each of the ten most frequently used corrective maneuvers or combinations of maneuvers. Suction was used more often in female than male patients (OR 1.68; 95% CI 1.04–2.69) and less often in patients in the HELP position than in those in the sniffing position (OR 0.32; 95% CI 0.14–0.68).

For the combination of BURP, suction, and blade change, the asphyxia subgroup compared with the trauma subgroup, and the HELP position compared with the sniffing position, met only a nominal threshold for significance (not reported). No other significant associations were identified.

### Multinomial analysis of single corrective maneuvers

Among cases in which a single corrective maneuver alone resulted in successful intubation and cell counts were sufficient, multinomial regression identified several significant associations. Age 85 years or older was associated with greater relative use of suction (RRR 2.02; 95% CI 1.02–3.99). Age 15 to 30 years (RRR 0.34; 95% CI 0.14–0.80) and age 61 to 85 years (RRR 0.52; 95% CI 0.32–0.86) were associated with lower relative use of BURP.

## Discussion

In this single-service retrospective observational study, a structured correction protocol used after a poor initial laryngoscopy view was associated with a high rate of subsequent intubation success. Among 604 cases in which at least one corrective maneuver was used, the primary endpoint was achieved in 578 (95.7%; 95% CI 98.4–99.3). First-pass success rate was 92.8% (2,255/2,431; 95% CI 91.7–93.8) and overall success 98.9% (2,405/2,431; 95% CI 98.4–99.3), comparing favorably with reported prehospital intubation outcomes [3].

Contemporary guidelines describe post-failure rescue well but provide limited evidence for structured corrective maneuvers applied before failed laryngoscopy is declared [5–14]. The present study extends this by evaluating a predefined, time-limited correction sequence embedded within a broader rapid sequence intubation standard operating procedure that also included a challenge-response checklist, routine bougie use, explicit attempt limits, reoxygenation when needed, and a predefined transition to rescue airway management. The findings therefore support the value of this structured, team-based sequence within a rescue-capable system rather than the isolated effect of any single maneuver [5, 36].

Suction and BURP were the most frequently used maneuvers and were commonly successful when used alone, but these rates are descriptive rather than evidence of comparative effectiveness. The two also differ in nature. Suction is essentially a binary decontamination step required to make the glottis assessable when blood or secretions obscure the view, whereas BURP and the other maneuvers align the airway axes and gradually optimize the laryngeal view [3]. Because a single maneuver is more likely to be chosen in less complex airways and multiple maneuvers in more difficult ones, the high success of individual maneuvers used alone probably reflects, in part, selection of less difficult cases rather than intrinsic superiority.

The lower success with more complex maneuver combinations probably does not indicate that these maneuvers are less useful. Rather, the need for multiple sequential corrections identifies patients with greater airway complexity, more severe physiologic derangement, challenging anatomy, or restrictive scene conditions, so that increasing complexity may serve as a practical marker of escalating airway difficulty.

These multivariable findings are best read as descriptors of the contexts in which correction was more often required rather than causal determinants. Male sex was associated with greater odds of correction, consistent with prior reports of more difficult laryngoscopy in men. Younger patients had lower odds than those aged 31 to 60 years, and internal medicine cases were less likely than trauma cases to require correction [18, 37, 38]. Operators from anesthesiology and intensive therapy required correction less often than those from emergency medicine or other specialties, consistent with greater airway expertise, whereas operator grade showed no clear association, possibly reflecting the standardizing effect of the protocol, checklist, and supervision [17].

The association between cricoid pressure and increased need for correction also warrants caution. Prior studies suggest cricoid pressure may increase intubation difficulty and does not reliably reduce aspiration [22]. In our cohort, it was infrequently used and, when present, associated with greater odds of correction, which may partly reflect selective use in patients perceived to be at higher aspiration risk. Similarly, the association with the HELP position more likely reflects confounding by indication, since HELP is commonly used when obesity or anticipated difficulty is already present [3].

Operationally, the finding that nearly two-thirds of correction cases were managed with a single maneuver supports the value of simple, rapidly executable corrective steps before escalation. Suction and BURP should remain central to training and cognitive aids in direct-laryngoscopy-based prehospital airway management, although their apparent yield reflects descriptive associations rather than proven comparative effectiveness [5, 36].

These findings are most applicable to direct laryngoscopy in a standardized physician-staffed HEMS. Video laryngoscopy is increasingly adopted, and our data do not support direct extrapolation of the protocol’s effect to it [39–41]. Video laryngoscopy may shift the problem from failure to visualize the glottis to failure to deliver the tube despite an adequate view, which likely requires its own correction strategy [40, 41]. A logical next step is prospective evaluation of a video-laryngoscopy-adapted protocol, particularly in “can see but cannot pass” scenarios. Notably, structured corrective sequences such as the 30-second drill remain in the standard operating procedures of leading physician-staffed services even after adopting video laryngoscopy, supporting their continued relevance [4, 42].

This study has several limitations. Its retrospective, observational design precludes causal inference. The high success rates cannot be attributed to the protocol alone, as case selection and other unmeasured factors likely contributed, and although we adjusted for operator specialty and grade, individual operator experience was not captured and could leave residual confounding. Registry entries were completed immediately after each mission, which minimized recall delay but remained susceptible to reporting and classification bias. In particular, the recorded initial Cormack-Lehane grade could not be reliably separated from the post-correction view, so we defined the cohort by documented corrective-maneuver use rather than by the initial grade. The sequence of maneuvers within combinations was not recorded, and several subgroup and combination analyses were based on small numbers, yielding unstable, exploratory estimates. The maneuvers are also heterogeneous (suction is a binary decontamination step, whereas the others progressively optimize the view), which limits direct comparison. Finally, the data come from a single-center physician-staffed HEMS operating across multiple bases in a highly standardized, direct-laryngoscopy environment, and the complete-case analysis could introduce bias if data were not missing at random, so the results may not generalize to systems with different staffing, training, airway devices, or rescue pathways.

## Conclusion

In this prehospital rapid sequence intubation system, a structured correction protocol used after a poor initial laryngoscopy view was associated with high subsequent intubation success. The predominance and high apparent yield of simple maneuvers, particularly suction and BURP, suggest that many episodes of difficult laryngoscopy were related to reversible and immediately solvable problems. Within a broader standardized airway management framework, these findings support using a brief, predefined, team-based optimization sequence before failed laryngoscopy is declared and rescue airway management is initiated.

## Supplementary Information

Below is the link to the electronic supplementary material.

Supplementary Material 1

Supplementary Material 2

## Data Availability

The data supporting the findings of this study are provided as supplementary material.

## Abbreviations

BURP: Backward-Upward-Rightward Pressure
BVM: Bag Valve Mask Ventilation
CI: Confidence Intervals
CL: Cormack-Lehane
FPS: First-Pass Success
HELP: Head-Elevated Laryngoscopy Position
HEMS: Helicopter Emergency Medical Service
HAA: Hungarian Air Ambulance Nonprofit Ltd
LMA: Laryngeal Mask Airway
MILS: Manual In-Line Stabilization
OR: Odds Ratio
RSI: Rapid Sequence Intubation
RRR: Relative Risk Ratio
SOP: Standard Operating Procedure
