# Supplementary material for: Structured correction after difficult direct laryngoscopy during prehospital rapid sequence intubation: an eleven-year physician-staffed helicopter emergency medical service registry study
Source: Int J Emerg Med. 2026 Aug 11;19:203. doi: 10.1186/s12245-026-01342-7 (PMC13464995; doi:10.1186/s12245-026-01342-7)
Supplement: Supplementary file 1 [file 12245_2026_1342_MOESM2_ESM.pdf]

RSI (Rapid Sequence Induction)

Emergency Anaesthesia and Intubation — HEMS Procedure

Hungarian Air Ambulance Nonprofit Ltd. · Air Ambulance Medical Working Group · Version 5 — 2024.05.06.

**Date of issue**	2024.05.06.
**Approved by**	Dr. Róbert Gebei, Medical Director
**Valid**	Until withdrawn
**Applies to**	Deployed medical personnel

Objectives

- To describe the indications for emergency anaesthesia.
- To describe how emergency anaesthesia is performed in adults and children.
- To describe what to do in case of failed laryngoscopy and intubation.

Background

- The Air Ambulance service performs approximately 300 RSIs per year (cumulative experience: more than 2,150 RSIs).
- During emergency intubation, the risk of hypoxaemia, aspiration, circulatory instability and cardiac arrest increases in proportion to the number of laryngoscopies. When intubating a critically ill patient, arrest is more frequent if induction is performed in the presence of hypoxia or hypotension, if there is no preoxygenation, and if the patient is elderly or overweight.
- The goal is for intubation to be successful and free of complications on the first look (DASH1a, Definitive Airway Sine Hypoxia/Hypotension on 1st Attempt).
- The guiding principle is that the first attempt must be optimized as much as possible. The essential elements of the procedure (see table) serve this purpose (among others), and therefore may not be deviated from.
- Behind non-DASH1a RSIs, unjustified SOP deviation(s) can often be identified.

Essential elements of the procedure

- Clear indications
- Telephone consultation before RSI
- Standardized patient positioning
- Clear roles and positions
- Routine preoxygenation, supplemented if needed with advanced oxygenation technique ± drug facilitation
- Before induction, circulatory optimization with fluid ± vasoactive support
- Standardized induction agent and relaxant selection and dosing
- Use of a checklist before induction
- Apnoeic oxygenation
- Unified communication during the laryngoscopy
- Routine bougie use
- Routine capnometry
- For a CL3–4 view, a 30-second procedure instead of blind attempts
- Standardized tube fixation
- Immediate correction of post-intubation hypotension with a bolus vasoactive agent
- Early post-intubation recruitment in case of hypoxic states
- Early switch to mechanical ventilation with lung- and neuroprotective settings
- Routine core temperature measurement after RSI

Preparation at the base

- Preparation for RSI begins at the base before the shift.
- At the start of the shift, an oxygen cylinder that is at least half full (101–200 bar, green) may remain in the airway bag. Based on a team decision, a cylinder at 51–100 bar (yellow) pressure may also be retained, but in this case the reserve cylinder must automatically be taken to every scene as well. At 50 bar or below (red), the cylinder must always be replaced — there is no room for economic considerations.
- The suction must be switched on and its vacuum-generating capacity tested by pinching/kinking the end of the rubber tubing. Suction strength is to be set to maximum. Leaks are usually caused by the connectors coming apart; this must be corrected. The rigid attachment must be connected.
- Both laryngoscopes must be checked; the blades must light up even when flexed.
- A bent bougie must be replaced. If it is not structurally damaged, it can regain its original shape by soaking it in hot water for a few seconds and then cooling it with cold water while hanging.
- Beyond checking the equipment, the morning checklist is at the same time a "rehearsal" for the RSI checklist. The brisk question-and-answer, avoiding repetition of the question, answering in full sentences, and extra acknowledgements set the pace for the on-scene checklist communication.

Dispatch to a (potentially) RSI-requiring patient

- Formation of a shared mental picture based on the dispatch information.
- As needed, calculation of sizes and drug doses (HELIHELP) and their cross-checking.

Indications and decision-making

Standard RSI

Indication	Strength
1. Inadequate or threatened airway	**STRONG**
2. Critical or impending oxygenation/ventilation disturbance	
3. Unconsciousness	
4. Any agitated head-injured patient	
5. Uncontrollably agitated patient requiring helicopter transport	
6. Anticipated further clinical course and treatment	**WEAK**
7. Humanitarian reasons	

Crash RSI

1. Patient requiring bag-mask ventilation due to a peri-arrest situation
2. Airway problem that cannot be controlled by simple means

- On-scene RSI requires a risk-benefit analysis adapted to the situation and the patient. The strength of the indication, the anticipated intubation difficulty, and the dynamics and time factor of the condition must all be assessed.
- For Standard RSI, optimal decision-making is aided by the telephone consultation.

RSI process

(Certain steps occur in parallel and/or are interchangeable.)

1. Non-specific, preliminary steps

- Basic airway management (manual manoeuvres, positioning, 2× NP, 1× OP, (continuous) pharyngeal suction).
- Oxygen administration (non-rebreather mask, 15 L/min), if needed by a dual method (mask + nasal cannula).
- SpO2, NIBP, ECG monitoring. If possible, the cuff opposite the (planned) vein.
- Consideration of starting invasive blood pressure measurement (per the Invasive Blood Pressure Measurement SOP).
- Securing at least one stable, sufficiently large-lumen vein (IO route), with a backflow preventer as needed.
- Insertion of a three-way stopcock if maintenance of narcosis by pump infusion, or a need for circulatory support, is highly likely.
- Packaging up to the chest, providing the best available thermal protection.
- Formation of a shared mental picture with the paramedic.

RSI flowchart

Exact reproduction of the original flowchart diagram (shown in landscape on the following page). Authors of the diagram: Sóti Á., Erőss A., Temesvári P.

RSI flowchart

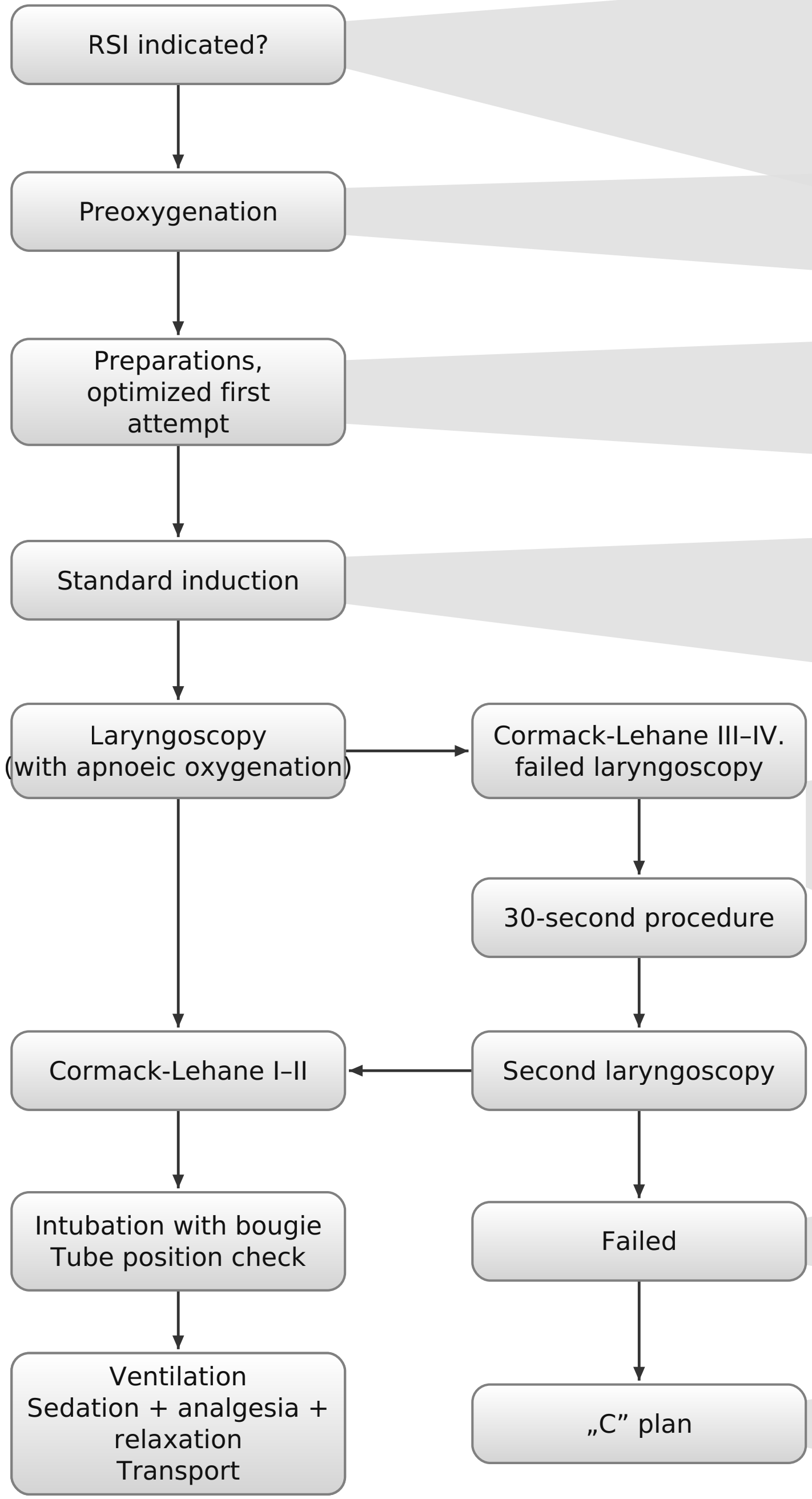

Indications

- inadequate or threatened airway
- respiratory failure – demonstrable or impending critical oxygenation disturbance
- unconsciousness
- patient severely agitated, struggling, with uncontrollable altered consciousness from head injury or other causes

Advanced oxygenation?

- dual oxygenation (cannula and mask)
- 4-handed assisted BMV
- mechanical NIV

Circulatory support?

- fluid bolus if not contraindicated
- preparation of push-dose adrenaline
- noradrenaline start considered

Induction agents

- ketamine 1 or 2 mg/kg, OR
- etomidate 0.15 – 0.3 mg/kg
- AND
- rocuronium 2 mg/kg

Failed laryngoscopy / 30-sec procedure

- patient position
- operator position
- suction
- BURP
- blade in-out manoeuvre
- blade change (usually longer, or McCoy)
- operator change

May be considered with 4-handed BMV:

- in case of C-L III and a “sensed” glottis, a single attempt by passing a bougie, guided by tactile stimulus (hold-up)

Failed intubation / „C” plan:

With 4-handed BMV:
- supraglottic device, OR
- surgical airway (immediately in case of CICO)

Sóti Á, Erőss A, Temesvári P.

2. Telephone consultation

- The provider temporarily steps back from the acute situation and structurally verbalizes the mental picture formed of the situation. This alone has an organizing effect on one's thinking. The heart rate also often decreases, resulting in a shift into a more ideal fine-motor performance range.
- The consultant — free from on-scene stress — assists with decision support, cross-checking (e.g. dose, device size), and identification of pre- and post-intubation specifics.
- Because of these, the consultation increases the safety of on-scene RSI, and is therefore mandatory before every standard RSI.
- The call must be made after the ABCDE assessment, in parallel with the preparations.
- In the case of crash intubation, the call is to be made after the intervention.

3. Patient positioning

- Full, 360° access all around.
- Placement at knee height — usually on a lowered ambulance stretcher.
- Adult not requiring cervical spine protection: external auditory canal at the level of the sternum, plane of the face horizontal. In a slim build this is achieved by elevating the occiput and tilting the head back (sniffing position); in an obese/heavily muscled patient by ramping starting from the mid-back (HELP).
- Cervical spine injury (suspicion): MILS in a true neutral head position, with occipital padding of a height depending on build.
- Severe, bleeding facial/skull injury: the body position that best supports maintenance of a spontaneous airway.
- In children, especially under 2 years of age, the neutral position is achieved by padding under the shoulders.
- Orthopnoeic, dyspnoeic patient: semi-sitting/sitting position.
- In every non-trauma patient, an elevated upper body is advantageous for oxygenation.

4. Roles and positioning of the providers

- Physician: watches the airway, the mask, the patient and the monitor, directs the helpers, and intubates. Kneels behind the patient's head (or positions at an optimal patient–operator height).
- In an RSI performed, out of necessity, in a position lying on the ground, the operator lies prone.
- Paramedic: selects the scene, performs the kit dump (lays out the equipment), directs the helpers, administers drugs (in the absence of a suitable helper), and assists with the intubation. Kneels on the physician's right side, at the patient's right shoulder.
- Helper 1: in trauma, performs MILS from the patient's left elbow side, without impeding mouth opening.
- Helper 2: administers drugs (if competent), performs BURP, holds the tube by hand and ventilates with the bag. Positioned at the vein or at the physician's left shoulder.

5. Preoxygenation

- Sufficiently long and effective preoxygenation is indispensable before intubating every patient with preserved circulation, as it delays the possible development of hypoxia during intubation.
- It requires a flawlessly patent airway (see point 1).
- Its basic device is a tightly fitting non-rebreather oxygen mask with rubber valves and a reservoir (non-rebreather mask, NRBM), which must be run at a flow of 15 L/min. Proper fit can only be achieved by holding the mask firmly onto the face; in this case the reservoir fills and empties in synchrony with breathing.
- In hypoxic patients, dual oxygenation must be performed via nasal cannula and NRBM using 15 L/min oxygen delivered in parallel.

- In non-traumatic respiratory failure, preoxygenation is to be performed with the following advanced techniques, preferably while maintaining the nasal cannula of the dual oxygenation:
  - Assisted, 4-handed reservoir-bag-mask ventilation with a PEEP valve.
  - Mechanical NIV support (the most effective method).

6. Pre-RSI sedation (facilitated preoxygenation)

- Its aim is the pharmacological facilitation of preoxygenation in an agitated patient with an unmanageable level of consciousness.
  - In case of pain: a major analgesic (0.5–1 mcg/kg IV fentanyl or 0.25–0.5 mg/kg IV ketamine), repeated. The advantage of fentanyl is that consciousness remains monitorable.
  - In case of hypoxic delirium caused by respiratory failure: 0.5–1 mg/kg IV ketamine, counting it into the induction dose (Delayed Sequence Intubation, DSI).
  - In case of head injury or other moderate agitation: 0.02 mg/kg IV midazolam is recommended; if significant agitation is present, or if repetition of midazolam is needed, sedation is to be continued with ketamine at a dose of 0.25–0.5 mg/kg (in hypovolaemia/hypotension the lower dose is to be given).
- Before/during sedation, the causes of the agitation (oxygenation, ventilation, perfusion disturbance, bladder distension, etc.) must also be treated in a targeted manner.

7. Premedication

- Routine premedication is not necessary before RSI.
- In consultation with the consultant, in selected cases 1 mcg/kg IV fentanyl may be given during the reading of the checklist to those haemodynamically stable (typically hypertensive) and easily preoxygenatable patients for whom the blood pressure surge possibly expected with laryngoscopy poses a serious risk (e.g. intracranial haemorrhage, non-compressible bleeding).
- Fentanyl premedication of a patient who has already received midazolam sedation is not recommended, as it increases the chance of a crash situation resulting from respiratory depression.

8. Circulatory optimization

- Even with circulation thought to be stable, post-intubation circulatory instability is to be expected if the heart rate is greater than 80% of the systolic blood pressure, i.e. if the shock index > 0.8.
- In the absence of a contraindication, it is recommended before every RSI to optimize the preload and correct hypovolaemia with 250 mL (child 10 mL/kg) crystalloid bolus(es).
- In case of circulation that remains unstable despite fluid bolus(es), before RSI it is recommended to:
  - Prepare push-dose adrenaline (1 mg diluted to 10 mL, then 1 mL of that diluted to a further 10 mL). This yields 10 mL of a 10 mcg/mL and 9 mL of a 100 mcg/mL adrenaline solution ready to hand.
  - Start noradrenaline in selected cases (e.g. sepsis, intoxication, TBI, other forms of shock), at a starting dose of 0.05–0.2 mcg/kg/min.
- Suitable for correcting post-intubation circulatory instability:
  - Adrenaline 20–100 mcg IV boluses (in children 1 mcg/kg).
  - Further fluid bolus.
  - Starting/increasing noradrenaline circulatory support.
  - Ventilation optimization (avoiding large VT and high PEEP).
  - Disease-specific therapy (e.g. thoracostomy).

9. Preparation of drugs and equipment (kit dump)

- Equipment needed for intubation on the fold-out bag (kit-dump module):
  - Drawn-up drugs: in standard dilution. If there is no prefilled syringe, the drugs must be drawn up and labelled per Appendix 1 (the relaxant first).
  - Laryngoscopes: tested in the morning; for a child the blade size agreed.
  - Bougie — Ch15: 6.5 mm ET or above; Ch10: 4–6 mm ET; Ch5: below size-4 ET (or optionally intubation without a bougie).
  - Tube and secondary tube: size agreed.
  - 20 mL syringe: with the plunger drawn out, fitted to the pilot balloon.
  - If available, use of a cuff pressure-measuring syringe to inflate the cuff (between 20–30 cm H_2O).
- Additional equipment around/on the kit-dump module, arranged clockwise:
  - Oxygen: one full cylinder and one reserve cylinder. Preferably a separate cylinder connected to the preoxygenation device and a separate one to the bag reservoir.
  - Tube-fixation tie: under the patient's neck.
  - Powered suction: beside the patient's head on the right, with the Yankauer rigid suction under the patient's head.
  - Monitor: on the patient's right side, in a place clearly visible to both the paramedic and the physician.
    - Capnograph connected and switched on (in case of failure, EMMA).
    - ECG and SpO_2 (with audible tone), and NIBP (with a 1–2 minute repeating measurement) on the patient. (If available, IBP checked.)
    - If possible, vein and NIBP cuff, and SpO_2 (adhesive) and NIBP cuff on different limbs. In case of an uncertain signal, an ear-clip sensor is recommended.
  - Bag with reservoir, valve, capnograph, and (appropriately sized) filter together. Ventilation mask separately.
  - Rescue devices (LMA, surgical airway): within reach.
  - Thoracostomy: ready for use, prepared, if needed.
  - Agreement on the post-ETI sedation strategy.

10. Reading the checklist

- The checklist (Appendix 2) is the team's final tool for verifying the technical and non-technical factors needed for a DASH1a intubation, i.e. for optimizing the first attempt.
- Reading the standard checklist ensures a dedicated preoxygenation time, allows the optimal placement of the required equipment to be checked, provides an opportunity to correct the patient position, allows a shared mental picture of the anticipated difficulties to be formed, and verbalizes the chosen C-plan and the post-intubation steps, thereby speeding up departure from the scene.
- For a crash situation, a shortened checklist — verifying key elements and equipment — is used.
- The physician checks the part above the thick line alone; the rest is checked together with the paramedic.
- When reading, the checklist must be put down/propped up (or held by a helper) so that both hands remain free for lifting the chin and fitting the mask to the face.
- The checklist is to be read aloud, word for word. The paramedic gives the defined short answer (avoiding answering in sentences and repeating the question) and points to/touches the given item. The physician moves on only after an answer, does not acknowledge the answer, and does not repeat it.
- On noticing a deficiency, the paramedic corrects with a "Wait!" signal and only gives the confirming answer after this.
- After a major interruption (e.g. vomiting), the checklist is to be continued by re-reading a few items.
- Checklisting is "well-invested time". Omitting either the standard or the crash checklist is a serious non-technical error. It is usually triggered by a mistaken judgement of the available time, the

worsening physiological deterioration alongside suboptimal early steps, and the false "illusion" of gaining time.

11. Induction

- Administration of the induction agent, then the muscle relaxant, in rapid succession, as a bolus, flushed in.

Induction agent		Note
**Standard**	ketamine 2 mg/kg IV/IO	Safe pre-hospital, the most stable from a circulatory standpoint, an agent that can also be given to head-injured patients.
**Alternative**	etomidate 0.3 mg/kg IV/IO	Preferred in extreme hypertension, phaeochromocytoma, high blood pressure threatening aneurysm rupture, thyrotoxic crisis, and severe coronary artery disease.

- In a haemodynamically unstable patient, the dose of the induction agent is to be halved.
- In a haemodynamically stable patient with a low GCS, the full dose is also recommended.
- In a severely haemodynamically unstable GCS 3 patient, and in a GCS 3 patient intoxicated with a sedative-hypnotic, the induction agent is to be omitted.

Muscle relaxant		Note
**Standard**	rocuronium 2 mg/kg IV/IO	Onset time: approx. 45–60 s. No contraindications.

12. Apnoeic oxygenation (ApOx)

- Passive oxygenation during the onset of the relaxant effect and during laryngoscopy, with oxygen led into the pharynx (15 L/min). It delays desaturation and is mandatory during standard RSI.
- Both the nasal (nasal cannula) and the nasopharyngeal method can be chosen (a cut oxygen extension tube led a few cm deep into an NP tube). The former is for good consciousness (e.g. burns), dual oxygenation or children, while the latter is rational in a patient already requiring an NP tube.
- Before laryngoscopy, in both methods the pharynx must be kept patent by lifting the chin.
- If only one oxygen source is available, the changeover is an extra task for the paramedic. In a single-cylinder bagged induction, however, there is no ApOx — enriching the bag with oxygen is the priority.
- During Crash RSI, ApOx is only an option with an already in-position nasal cannula/NP tube.

13. Post-induction bag ventilation

- After induction, assisted, then controlled, 4-handed bag-mask ventilation of the patient is to be performed in the following cases:
    - Every patient in respiratory failure who is difficult to oxygenate (exception: suspicion of tension PTX).
    - Suspicion of severe metabolic acidosis requiring maintenance of ventilation.
    - A child judged by the provider to be of primary-school age or younger.

14. Laryngoscopy and obtaining an adequate view

- Laryngoscopy begins only with a slack jaw: approx. 45–60 s later.
- Although there is a strong urge to bring the laryngeal inlet into view immediately, gradual view-gaining with fine movements is more advantageous and is expected even under stress: identify the base of the tongue → identify the epiglottis → fit the blade into the vallecula → expose the laryngeal inlet.
- The provider should aim for the minimally necessary view and not for a Cormack-Lehane I grade.
- The physician optionally verbalizes reaching the epiglottis and the glottis, and mandatorily verbalizes the obtained view. The paramedic signals an SpO2 falling below 95% and prepares for

the 30-second procedure. This close communication helps maintain the shared mental picture during the laryngoscopy.

- Intubation is recommended only with a Cormack-Lehane I–II grade. If the glottis cannot be brought into view (Cormack-Lehane III–IV grade), the 30-second procedure is to be followed.
- With a Cormack-Lehane II view, the more of the glottis that is visible (ideally at least 50%), the easier the passage of the bougie/tube. BURP may also be considered for a "weak" Cormack-Lehane II view (especially if only the arytenoid cartilages are visible).

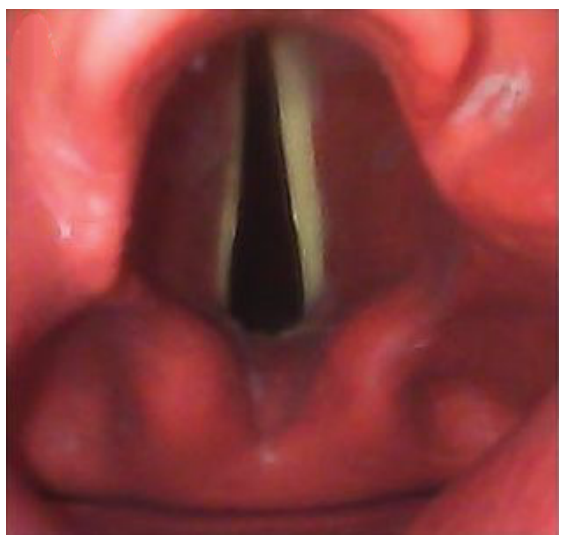

Cormack-Lehane I
The glottis is visible in its entirety (from the apex = anterior commissure to the arytenoid cartilages).

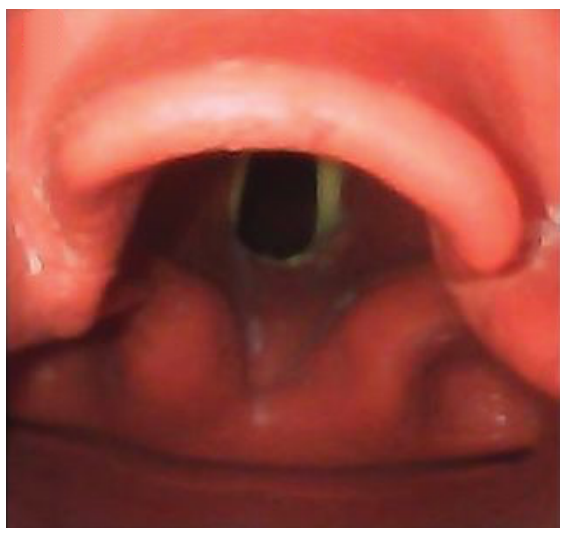

Cormack-Lehane II
The glottis is partly visible (here approx. 40%) or not visible, but the arytenoid cartilages still are.

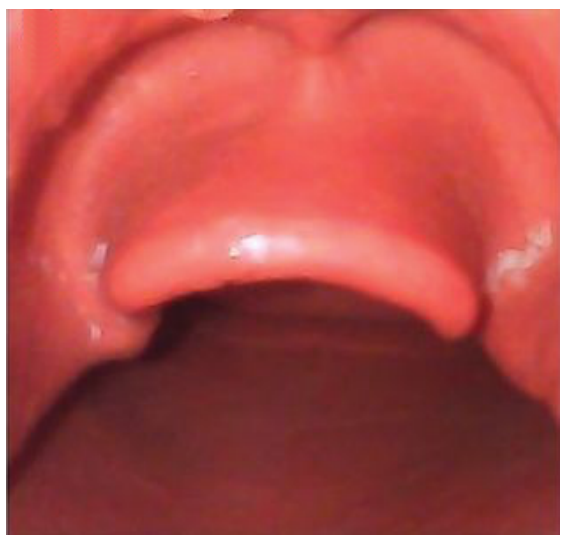

Cormack-Lehane III
Only the epiglottis is visible.

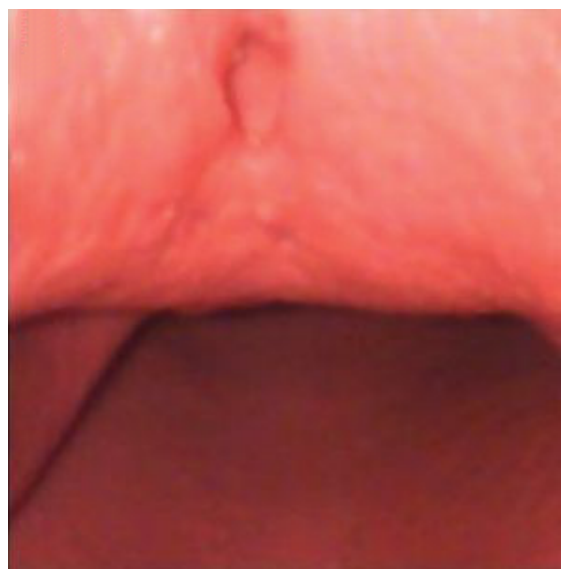

Cormack-Lehane IV
The epiglottis is not visible either.

15. Passing the bougie and tube

- The use of the bougie increases the rate of successful first-attempt passage of the tube even with an adequate view, and is therefore mandatory during every RSI.
- Handling of the bougie and the tube is teamwork carried out according to a strict script.
- The paramedic gives the bougie into the physician's right hand at the one-third point; as soon as the bougie is in the physician's hand, the paramedic releases it.
- One must aim to pass the bougie into the trachea without touching the surrounding structures, on the first try and only 3–5 cm. Tracheal clicking may perhaps be felt. Communication: "bougie has passed through the glottis".
- The paramedic threads the tube onto the bougie, then firmly communicates ("I have the bougie") when they have grasped the protruding end.
- The physician releases the bougie and grasps the tube. Communication: "I have the tube".
- When passing the threaded tube, one must consciously watch for the cuff to just disappear below the glottis, preventing deep (main-bronchus) placement. The black marker lines help with this. Communication: "cuff has passed through the glottis".
- If the tube gets held up at the glottis, after slight withdrawal it must be rotated 90° counterclockwise, passed through this way, then rotated back and pushed into position. If this fails, a smaller tube must be used.
- The physician holds the passed tube (with the bougie inside it) in place, while the paramedic withdraws the bougie and inflates the cuff using the pressure-measuring syringe. During inflation the paramedic palpates the pilot balloon (elastic but not tense), while the physician watches for any herniation of the cuff or displacement of the tube. If so, they signal it and advance the tube deeper.
- Following this, the paramedic connects the breathing circuit, begins ventilation and verbalizes the EtCO2 value.
- The view is to be maintained until capnography confirms successful intubation.
- In case of a low and further decreasing, or 0 or non-measurable EtCO2, the tube must be removed and, after re-ventilation, preparation must be made for a repeat laryngoscopy supplemented with 30-second elements, or for the C-plan.

- While withdrawing with the blade, the physician verbalizes the tube depth (cm) at the corner of the mouth. Normal tube depth is usually 21–23 cm in an adult man and 20–22 cm in an adult woman. A value that differs significantly from this should raise suspicion.

16. Checking and correcting the tube position

- The endotracheal position is confirmed by an interpretable capnograph result.
- An endobronchial position must be excluded by 4-point auscultation (bilateral apical and basal).
- In case of a tube advanced too deep, based on both auscultation and cm, stepwise withdrawal (by 1 cm in adults, 0.5 cm in children) with continuous auscultation is generally recommended. For this manoeuvre the cuff need not be deflated, and ventilation must be continued. The final depth must again be verbalized numerically.

17. Tube fixation

- Immediately after intubation, the tube is held by the paramedic, then by the delegated helper, at the defined (cm) depth, propped against the corner of the mouth.
- After checking the tube position (and performing the urgent task(s)), the paramedic switches to tie fixation: with the tie ends led under the occiput (ear), a simple knot is made at the defined cm on one side and then the other side of the tube; then the ends are looped back and the whole thing is fixed with a double knot under tension. Compressing the neck veins and narrowing the tube lumen (typical especially with a thin tube) is to be avoided.
- Regardless of fixation, the tube must be held with one hand at the defined depth (cm) whenever the patient is moved. An exception is the mechanically ventilated patient in whom the ventilator and breathing circuit are fixed to the bed; in this case it is enough to observe the tube, and it need not be held.
- The fixed tube must be checked regularly to see whether the correct depth figure is visible at the teeth. At every patient movement, the depth or any displacement must be controlled by continuous visual monitoring, making sure that nothing can pull/push the tube.

18. The 30-second procedure

- A series of manoeuvres aimed at improving a Cormack-Lehane III–IV view, which the physician and paramedic carry out together.
- During the manoeuvre, the paramedic's task is to watch and announce aloud the saturation and the elapsed time (the physician's perception of the outside world and of time may be distorted while focused on the task).
- Its elements:
  - Correction of the patient's position: head in a true neutral position (occipital padding), neck perpendicular to the shoulder girdle, jaw slack; in a non-trauma case, correct sniffing position/ HELP.
  - Correction of the physician's position: lowering/rising to the ideal operator–patient height, kneeling on both knees; in a patient lying on the ground, lying prone or lifting the patient to stretcher height.
  - Airway suction: suctioning secretions/foam ball obscuring the view with a Yankauer rigid suction (not with a suction catheter). After lifting up the glottis, suctioning secretions pooled at the floor of the pharynx that do not obscure the view is not necessary, since during intubation the main goal is DASH1a.
  - BURP: external manipulation of the thyroid cartilage with pressure applied Backward, Upward and to the Right; the 2-person technique with a helper is best.
  - Blade in-out manoeuvre: full insertion of the laryngoscope blade, then slow withdrawal and identification of the epiglottis that flips down or of another structure. If needed, positioning of the patient's head with the right hand (except in MILS).
  - Blade change: more often larger than smaller; occasionally a McCoy blade is needed.
  - Operator change: only if there is a cause of failure that cannot be otherwise explained, and a provider authorized to intubate is present.
- The procedure means considering all elements, but the order can be adapted to the situation:
  - In case of secretions worsening the view, suction is logically the first choice.

  - In case of a Cormack-Lehane III view, applying BURP first is rational.
  - In case of a Cormack-Lehane IV view, it is worth starting with checking the patient position, then the blade in-out.
- If, even after all elements of the 30-second procedure, only a Cormack-Lehane III grade can be achieved, passing the bougie into the trachea may be considered with a single attempt.
  - The curved end, kept in the midline, must be directed behind the epiglottis.
  - During passage, tracheal clicking may be felt, but only the hold-up of the cautiously advanced device provides definitive confirmation (at approx. 30 cm in an adult).
  - After the hold-up, but before threading the tube, the bougie must be withdrawn several cm so that manipulation does not cause bronchial injury.
  - If there is no hold-up even at 40 cm (proportionally earlier in a child), the bougie is in the oesophagus. Threading the tube is forbidden, and no further attempt is permitted. The C-plan must be initiated.
- If, even after all elements of the 30-second procedure, only a Cormack-Lehane IV grade is seen, the C-plan must be initiated automatically. A "blind" attempt is forbidden.

19. Failed intubation

- Intubation has failed if, after the 30-second procedure and reoxygenation, the glottis cannot be brought into view on a second attempt either (Cormack-Lehane III–IV grade), or the correct position cannot be confirmed with $EtCO_2$.
- Based on careful judgement, a third attempt is also acceptable if, after the second, a problem correctable with one of the elements of the 30-second procedure was identified.
- An important element of 4-handed bag-mask reoxygenation is the use of simple airway aids: with an existing NP, supplement with an OP; if neither is inserted, begin with the OP.
- In case of failed intubation, during 4-handed bag-mask reoxygenation the team must weigh which C-plan (LMA or surgical airway) to choose.
- If oxygenation can be ensured with an LMA or with bag-mask ventilation, a further telephone consultation should be considered regarding the choice of the definitive C-plan solution.

A. Laryngeal mask (LMA)

- The equipment contains a single-use second-generation LMA.
- Size selection is aided by the kg range on the pilot balloon.
- Insertion can be performed, per individual preference, with a fully deflated, wrinkle-free cuff or with a partially inflated cuff.
- The two-person technique can help: a helper lifts the jaw with both hands and opens the mouth, while the physician inserts the device. An experienced provider may deviate from this.
- Inflation of the cuff is first to be done with the maximum volume indicated on the pilot balloon.
- A good seal is indicated by the absence of a leak, the chest rising on insufflation, and a normal $EtCO_2$ waveform.
- Fixation of the device is done similarly to the fixation of the endotracheal tube.
- If transport with the LMA is planned, after reoxygenation a gastric tube must be passed through the gastric port. The maximum tube diameter is indicated by the "Max Gastric Tube" marking.

B. Surgical airway management

ON-SCENE INDICATIONS FOR CRICOTHYROTOMY

A. CICO situation: the patient cannot be intubated and cannot be oxygenated by any means.

B. LMA oxygenation is partially effective, the patient's condition is gradually deteriorating.

C. LMA is effective for the moment, but will no longer be safe for transport:

- airway burn
- chest trauma
- acute asthma (or high airway pressure expected for another reason)
- high risk of aspiration

D. Primary cricothyrotomy: a patient requiring intubation in whom laryngoscopy/LMA is not a realistic option:

- a face, mouth, jaw burned rock-hard
- a patient with a fixed jaw after oral surgery
- a casualty trapped by the face but with a free neck, in a peri-arrest state

1. Continuation of the already-started oxygenation techniques is recommended.
2. Positioning: reclined head, neck lifted forward (head-block under the shoulder, or drawn up on a stretcher with the head hanging). MILS is to be sacrificed.
3. Palpation of the larynx and the cricothyroid ligament (laryngeal handshake).
4. In an easily palpable case, piercing the ligament with a horizontal blade.
5. In a hard-to-palpate/non-palpable case (e.g. bulky soft tissue, burn, scar, oedema, subcutaneous emphysema, deformity):
    - a. making an approx. 8–10 cm midline aiding incision

    - b. exposing the area by pulling apart the skin and soft tissues (e.g. with a finger, with a haemostat)
    - c. palpating the ligament
    - d. piercing the ligament with a horizontal blade (the edge of the blade facing away from the provider to avoid injury to the operator)

6. Rotating the scalpel 90° so that the tip of the blade faces caudally, then opening a gap by pulling slightly toward oneself.
7. Introducing a Ch10 bougie alongside the scalpel into the trachea, on the side of the scalpel opposite to the provider.
8. Removing the scalpel.
9. Introducing a 6.0 mm tube over the bougie, until the cuff disappears into the lumen.
10. Removing the bougie.
11. Inflating the cuff.
12. Ventilation, capnograph-guided.
13. Checking the tube position in the usual way.
14. Fixing the tube with the usual knotting, avoiding neck strangulation.

- The striving for sterility is instinctive, but when immediate intervention is needed (A, B, D/3) it is forbidden to prolong the life-saving with disinfection (without waiting for it to take effect it would only be a show activity anyway). With a smaller time factor (C, D/1, D/2) disinfection is possible.
- The hardest part is the decision-making. For the sake of "mental commitment", before an anticipated difficult RSI it is recommended to verbalize the potential cricothyrotomy, prepare the equipment, palpate the neck and decide on the site of the incision, and disinfect the area.
- In case of difficult visualization (soft tissues lying together despite bleeding or a longitudinal incision), the scalpel must be withdrawn and the opening identified with a finger, then the bougie introduced alongside the finger. During the blind technique, one must rely on the fine sensing of the fingertip.
- Bleeding noticed and persisting after successful surgical airway management can be controlled with direct pressure, under-suturing, a haemostat, and haemostatic dressing.

20. Ventilation and relaxation

- After airway management, bag ventilation, then mechanical ventilation as soon as possible, is warranted.
- Initial settings: IPPV mode, FiO2 1.0, PEEP 5, VT 6–8 mL/kg, rate 10–16/min, then adjusted EtCO2-guided (default target: 35–40 mmHg).
- In a patient with respiratory failure, alveolar recruitment is to be performed on scene if the circulation is sufficiently stable:
  - With the ventilator: an inspiratory-hold manoeuvre (Insp Hold button) at 20 PEEP for a maximum of 40 s, then setting PEEP to 5–10 (or raising it above the original value).
  - With the bag: with the PEEP valve closed, approx. 1 minute of ventilation with larger volumes and short expiratory pauses, then setting PEEP to 5–10 (or raising it above the original value).
- Identification and resolution of the cause of non-resolving or unexpectedly occurring hypoxia can be aided by the structured DOPES approach:
  - D — displacement — the tube sliding too deep, or slipping out
  - O — obstruction — partial or complete obstruction, e.g. due to kinking, secretions (tube, filter, breathing circuit)
  - P — pneumothorax — a large PTX, or one becoming tension
  - E — equipment — equipment problem, anything from the oxygen source all the way to the tube cuff
  - S — stomach — a ventilation problem due to gastric distension
  - S — stack breath — dynamic hyperinflation
- In the absence of a contraindication, maintaining an elevated head-end/HELP is recommended even after intubation.
- Mechanical ventilation must be continued without interruption until handover, avoiding suboptimal manual bagging. For this, if needed, one's own ventilator must be taken out of the helicopter.
- In a clearly low-time-factor condition, mechanical ventilation is preferred already on scene.

21. Maintenance of narcosis

- Setting up the post-intubation narcosis plan as early as possible — in a stable patient already before induction — is necessary. In a haemodynamically unstable patient, after successful intubation the sedative strategy must be decided as soon as possible, and it must also be started immediately. The most common signs of suspected light anaesthesia are tearing (the simplest), tachycardia and blood pressure rise, and an unexpected EtCO2 rise signalling an increase in cardiac output.
- A split EtCO2 waveform, coughing, respiratory movement, and dyssynchrony indicate the waning relaxant effect.

Drug	BOLUS	PUMP INFUSION
ketamine	0.25 mg/kg IV (adult 20–30 mg), every 5–10 min	1–5 mg/kg/h IV*
propofol	No	1–5 mg/kg/h IV
midazolam	0.02 mg/kg IV (adult 1–2 mg)	Not primarily**
fentanyl***	1–2 mcg/kg IV, every 20–30 min	Not primarily**
morphine***	0.025–0.05 mg/kg IV, every 30–40 min	Not primarily**
etomidate	No	No

* standard dilution: 500 mg ketamine diluted to 50 mL
** continuation of a dosing started in hospital may be considered
*** not recommended on their own, only as a supplement to the agents above them

22. Preparation for transport

- Continuous SpO2, EtCO2, ECG, NIBP/IBP monitoring.

- Passing a core-temperature probe.
- Bundled routing of the monitor cables toward the right shoulder.
- Consideration of a warming pad.
- Closing the thermal-protective packaging, correcting any sensors/cables that fall off in the process.
- Keeping the venous access free by punching a hole in the foil and threading the infusion(s) through.
- In case of neck trauma, applying/reapplying the cervical collar.
- Head-block fixation in every patient.
- The Post RSI checklist (see Appendix 2) helps to check the most important elements.

Crash RSI

- Induction and intubation take place as soon as possible.
- If bag-mask ventilation is needed until induction, it must be performed with airway aids (2× NP, 1× OP), reservoir (100%) oxygen and a four-handed technique (the physician lifts the jaw with both hands and presses the mask to the face, the helper squeezes the bag).
- The Crash RSI checklist is to be used (see Appendix 2), which may not be omitted even for the sake of saving time. Despite the high time factor, checking the critical elements is well-invested time.
- Induction is performed according to the circulation/consciousness — but in any case with a relaxant.

Paediatric RSI

- Up to primary-school age (especially in infancy and toddlerhood), a few elements of RSI are modified.
- The indications are the same, but the risk/benefit analysis will more often argue against RSI. The telephone consultation gains special emphasis.
- For the preoxygenation and intubation of infants and toddlers, a neutral head position must be ensured by padding under the back and shoulders.
- Up to primary-school age, opening the airway is done with an OP tube, positioning and chin lift. If this is not enough, or continuous suction is needed, passing an NP tube (one size smaller than the calculated ET size) may also be considered.
- For the drug doses, the body weight must be obtained from the parent. If it is not known, then between 1 and 10 years the formula weight (kg) = (age + 4) × 2 must be used.
- Blade, bougie, tube, bag, filter, face mask and LMA: to be prepared in the size appropriate for age. The sizes of certain devices can be calculated between 1–10 years of age:
  - cuffed tube size (mm) = (age / 4) + 3.5
  - tube length at the teeth (cm) = (age / 2) + 12
  - bougie: 15 Ch = tube of 6.5 mm or above, 10 Ch = tube of 4–6 mm, 5 Ch = tube below 4 mm (it is recommended to pass the bougie through the tube to verify that the sizes match)
- An atropine pack must be within reach; under 1 year of age it must also be drawn up. It is for treating (the rare) bradycardia of non-hypoxic origin (0.02 mg/kg, but at least 0.1 mg IV).
- The use of a dose card or the table on the checklist increases the accuracy of calculation and size selection. The latter deviates from the formula above 7 years of age, because here the body weight is typically higher. Use of the formula is also acceptable, but the table values are more accurate.
- Because of the faster desaturation, bag-mask ventilation is recommended between induction and laryngoscopy. Proper hand position, head–neck position, FiO2 1.0 and the use of low pressures are important. In case of prior/post-induction hypoxia, laryngoscopy should begin only after one minute of effective ventilation (ideally at 100% SpO2).
- Apnoeic oxygenation can be performed with a nasal cannula.
- Use of the smallest-size (5 Ch) bougie is optional; this requires a prior physician–PM decision.
- Too-tight tube fixation can narrow the lumen. This must be actively watched for when knotting, and must also be borne in mind in a child taken over from someone else who can be ventilated with a high peak pressure.
- We are forced into surgical airway management extremely rarely in children. Even in case of intubation failure, the LMA is usually a good solution. Of course, in certain conditions, in the absence of another solution (CICO), we are forced into surgical airway management.
- The technique of cricothyrotomy above 8 years of age matches the adult method, but requires finer movements and a proportionally smaller tube.
- Below 8 years of age, performing a tracheostomy is recommended with the known scalpel–bougie technique at the level of the 1st and 2nd tracheal rings. Its technique differs in part from cricothyrotomy: after a longitudinal skin incision, a longitudinal incision is made with the scalpel on

the tracheal cartilages, spanning a maximum of two rings; then, having entered the lumen, the bougie is introduced along the scalpel, and there is no need to rotate it. Following this, a tube appropriate to the body size is threaded on in the usual way.

- In infants, accurate measurement of EtCO2 is often uncertain because of the dead space of the breathing circuit, so one must try to ventilate close to the physiological respiratory rate and minute ventilation.
- Passing a gastric tube is necessary, especially if bag-mask ventilation was also performed.
- Technical limits of the Oxylog 3000+: VT 50 mL, RR 60/min. For volumes smaller than this, manual ventilation is necessary. The dead space of the adult multi-use circuit (to the valve) is 35 mL.
- In case of a progressive EtCO2 rise, to reduce the dead space, bypassing the mechanical circuit (manual ventilation) and, as a last resort, removing the filter are options.
- Use of a paediatric (EH) breathing circuit is recommended below approx. 40 kg (below 250 mL VT).

Documentation

- The data needed for the RSI audit are collected by HEMSDok; completing these is mandatory.
- Indication: if more than one is present, the one playing the greatest role in the decision must be coded.
- Start time: the time of starting the physician's part of the checklist.
- End time: the time of completing the tie fixation of the tube or, if this was pushed to later because of a more urgent task, then the time of the tube position check.

Additional documentation definitions (per the source):

- Aid, crash, telephone, ApOx, preoxygenation, patient position: as applicable.
- View of the first laryngoscopy: as applicable. Only a glottis visible in its entirety, starting from the anterior commissure, qualifies as a Cormack-Lehane I grade.
- 30-second procedure: as applicable, one or more.
- Independently of the elements of the 30-second procedure, it is to be interpreted as a second attempt if the laryngoscope passes between the teeth a second time. A BURP, suction, blade in-out, or position correction that leads to a solution in a short time does not qualify as a multiple attempt.
- Second 30-minute [30-second] procedure: as applicable.
- Complication: as applicable. If there was none, the "there was none" field is to be marked.
- Blade, tube, bougie size, tube depth and C-plan: as applicable.

References

1. Crewdson K, et al. Best practice advice on pre-hospital emergency anaesthesia & advanced airway management. Scand J Trauma Resusc Emerg Med. 2019;27:6.
2. Sóti Á, et al. Rapid Sequence Induction in the practice of the Hungarian Air Ambulance Nonprofit Ltd., results of a six-and-a-half-year prospective study. Aneszt. Int. Th. 2019;49:16-25.
3. Higgs A, et al. Guidelines for the management of tracheal intubation in critically ill adults. Br J Anaesth. 2018;120:323-52.
4. De Jong A, et al. Cardiac Arrest and Mortality Related to Intubation Procedure in Critically Ill Adult Patients: A Multicenter Cohort Study. Crit Care Med. 2018;46:532-9.
5. Park L, et al. Systematic review and meta-analysis of first-pass success rates in emergency department intubation: Creating a benchmark for emergency airway care. Emerg Med Australas. 2017;29:40-47.
6. Driver B, et al. Effect of Use of a Bougie vs Endotracheal Tube and Stylet on First-Attempt Intubation Success Among Patients With Difficult Airways Undergoing Emergency Intubation. JAMA. 2018;319:2179-89.
7. Driver B, et al. The Bougie and First-Pass Success in the Emergency Department. Ann Emerg Med. 2017;70:473-478.e1.
8. Natt BS, et al. Strategies to improve first attempt success at intubation in critically ill patients. Br J Anaesth. 2016;1117 Suppl 1:i60-i68.
9. Bernhardt M, et al. The First Shot Is Often the Best Shot: First-Pass Intubation Success in Emergency Airway Management. Anaesth Analg. 2015;121:1389-93.
10. Eross A, et al. Apneic preoxygenation without nasal prongs: the "Hungarian Air Ambulance method". Scand J Trauma Resusc Emerg Med. 2016;24:5.
11. Haag AK, Tredese A, Bordini M, Fuchs A, Greif R, Matava C, Riva T, Scquizzato T, Disma N. Emergency front-of-neck access in pediatric anesthesia: A narrative review. Paediatr Anaesth. 2024 Jun;34(6):495-506.

Appendices

Appendix 1: Dilution and labelling of drugs used during RSI

Active substance	Label	Syringe	Concentration	Preparation of one syringe
Ketamine		2 × 10 mL	10 mg/mL	2 mL ketamine + 8 mL NaCl 0.9%
Rocuronium		(2×) 10 mL	10 mg/mL	drawn up undiluted, all together
Etomidate		10 mL	2 mg/mL	drawn up undiluted
Midazolam		10 mL	1 mg/mL	2 mL midazolam + 8 mL NaCl 0.9%
Fentanyl		10 mL	50 mcg/mL	drawn up undiluted

Label fields: Ketamine .... mg/mL · Rocuronium .... mg/mL · Etomidate .... mg/mL · Midazolam .... mg/mL · Fentanyl .... micrograms/mL

Appendix 2: RSI checklist

Hungarian Air Ambulance Nonprofit Ltd., Version: 5.3 (2024.05.05.)

(DR = doctor/physician; PM = paramedic. The item is read aloud, the responder gives the short response shown at right.)

Standard RSI checklist

Item	Responder	Response
Shadow (checklist) — in order	DR	in order
Patient position (HELP/sniffing/occiput)	DR	in order
Upper airway …	DR	maximally open
TRAUMA — cervical collar … (if present)	DR	removed
MILS … (if needed)	DR	in order
Two-handed fitted 100% mask …	DR	filling
Advanced oxygenation? (if SpO2 < 98%)	DR	considered
Vein …	DR	patent
Cuff (BP) …	DR	on the other arm
Fentanyl needed? … mcg, i.e. … mL	DR	administered
Diluted adrenaline drawn up?	DR	considered
Content of the phone call …	DR–PM	…
Oxygen pressure … bar	PM	…
Second cylinder on the bag, open	PM	yes
Suction and tie under the head	PM	yes
Saturation? Pulse? Blood pressure?	PM	…
Blood pressure on repeating measurement	PM	yes
Capnograph picking up a signal	PM	yes
Bag–capnograph–filter together + mask	PM	yes
Bougie	PM	yes
Ketamine … mg, i.e. … mL	PM	… mL
Rocuronium … mg, i.e. … mL	PM	… mL
Tube with syringe	PM	size …, on it

CHILD		
Alternative tube	PM	size …
Blades	PM	size … and …
Appropriately sized bougie	PM	yes
Mask on the bag	PM	yes
NP/OP prepared	PM	yes
C-plan — size … LMA / surgical airway will be used	PM	to hand
Thoracostomy … (if needed)	PM	taken out
My own position in order?	PM	yes
Prepared for ApOx?	PM	yes
Post-ETI analgesia and sedation plan	PM	agreed
Induction	PM	… h … min

30-second procedure (checklist)

Patient position · Intubator position · Suction · BURP · Blade in-out · Blade change · Intubator change

Post RSI checklist

Tube depth · EtCO2 set · Tube fixation · VT 6–8 mL/kg · Analgosedation · Vasoactive agent? · Oxygen open? · Core temperature measurement · Relaxant, Sedation · Warming pad? · Thoracostomy? · Cable bundling · Recruitment? · FiO2 reduction? · Loose collar · Elevated head-end

Paediatric dosing table (checklist)

Age / kg	ROC / KET	ROC / KET (mL)	Cuffed tube	Length	LMA	Bougie
½ / 7	14 mg	1.4 mL	3.0	12 cm	1½	(5 Ch)
1 / 10	20 mg	2.0 mL	3.5	12.5 cm	1½	(5 Ch)
2 / 12	24 mg	2.4 mL	4.0	13 cm	2	(5 Ch)
3 / 14	28 mg	2.8 mL	4.0	13.5 cm	2	(5 Ch)
4 / 16	32 mg	3.2 mL	4.5	14 cm	2	10 Ch
5 / 18	36 mg	3.6 mL	4.5	14.5 cm	2	10 Ch
6 / 20	40 mg	4.0 mL	5.0	15 cm	2½	10 Ch
7 / 23	46 mg	4.6 mL	5.0	15.5 cm	2½	10 Ch
8 / 26	52 mg	5.2 mL	5.5	16.5 cm	2½	10 Ch
9 / 30	60 mg	6.0 mL	5.5	16.5 cm	3	10 Ch
10 / 34	68 mg	6.8 mL	6.0	17 cm	3	15 Ch
12 / 40	80 mg	8.0 mL	6.5	18 cm	3	15 Ch
14 / 50	100 mg	10 mL	7.0	19 cm	3	15 Ch

(ROC/KET column: rocuronium and ketamine doses coincide numerically in this table, in mg and mL respectively.)

Crash RSI checklist

Item	Responder	Response
Shadow (checklist)	DR	in order
Patient position …	DR	in order
Cervical collar … (if present)	DR	removed
Bag–capnograph–filter–mask	PM	yes

Oxygen on the bag, open	PM	yes
Suction	PM	yes
Blade size …	PM	size …
Bougie	PM	yes
Tube size …	PM	size …
Syringe	PM	on it
Infusion running	PM	yes
Rocuronium … mg, i.e. … mL	PM	… mL
My own position	PM	in order
Induction … at … h … min		

Paediatric dosing table (Crash RSI checklist)

Age / kg	ROC (mL)	Cuffed tube
½ / 7	1.4 mL	3.0
1 / 10	2.0 mL	3.5
2 / 12	2.4 mL	4.0
3 / 14	2.8 mL	4.0
4 / 16	3.2 mL	4.5
5 / 18	3.6 mL	4.5
6 / 20	4.0 mL	5.0
7 / 23	4.6 mL	5.0
8 / 26	5.2 mL	5.5
9 / 30	6.0 mL	5.5
10 / 34	6.8 mL	6.0
12 / 40	8.0 mL	6.5
14 / 50	10.0 mL	7.0

Revision history

Version 5 — PRESENT DOCUMENT (2024.05.06.)

Title: RSI (Rapid Sequence Induction). Emergency anaesthesia and intubation.
Authors: Dr. Attila Erőss, Dr. László T. Hetzman, Dr. András Petróczy, Dr. Ákos Sóti, Dr. Péter Temesvári, Dr. Róbert Gebei, Dr. Zsolt Dubóczki, Dr. Ákos Jászkuti, Balázs Bondor, Mátyás Solténszky, Attila Haja

Changes:

- Use of succinylcholine discontinued
- Two-oxygen-source requirement
- The CPAP and oxygen-reservoir-valve-mask-PEEP system removed
- Noradrenaline dose modified
- EasyCap removed
- Wording clarifications
- Phrasing refined
- Bougie grip clarified
- Tube depth clarified and tube grip modified
- Introduction of cuff pressure measurement
- Surgical airway description clarified
- Paediatric ventilation and cricothyrotomy modified
- Documentation: a second laryngoscopy if the blade passes between the teeth again
- Checklist: the ventilation mask is included

Version 4 — (2020.12.01.)

Title: RSI (Rapid Sequence Induction). Emergency anaesthesia and intubation.
Authors: Dr. Attila Erőss, Dr. László T. Hetzman, Dr. András Petróczy, Dr. Ákos Sóti, Dr. Péter Temesvári

Changes:

- New format, revision history
- Condensation, rephrasing in places
- Newly presented and supplemented algorithm
- DASH1a mentioned as a quality indicator
- New chapter: preparation at the base and dispatch to an RSI-requiring patient
- Clarification of indications
- Refinement of positions, introduction of occipital padding
- Clarification of provider roles
- Recommendation of dual oxygenation
- Mechanical NIV mentioned among the advanced oxygenation techniques
- Mention of facilitated preoxygenation, expansion of the drugs with fentanyl, with mention of advantages
- Circulatory optimization elaborated
- Optimal checklisting detailed
- Induction dose reduction clarified
- For the succinylcholine contraindication, the non-fresh burn within 24 hours clarified
- Indications for bagged induction defined
- Laryngoscopy, bougie/tube passage and tube fixation elaborated in detail
- BURP recommendation for a "weak" CL II
- Description for the case of a tube advanced too deep

- 30-second procedure clarified: order adapted to the situation, recommendation for handling pharyngeal secretions that do not obscure the view
- Tactile CL III attempt clarified
- Elaboration of the use of the laryngeal mask
- Surgical airway: expansion of indications, scalpel-(finger)-bougie-tube technique, omission of the dilator
- Mention of post-intubation recruitment
- Preference for early post-intubation mechanical ventilation
- Possibility of a reduced-dose rocuronium
- More detail on maintenance of narcosis, ketamine pump-infusion dosing
- Expansion of preparation for transport
- Omission of the Broselow tape
- Paediatric tube size age / 4 + 3.5
- Recommendations regarding dead space in the ventilation of small children
- Documentation section, clarification of definitions
- Updated checklists, with a new "Post RSI checklist"

Version 3 — 2016.12.20.

Title: RSI (Rapid Sequence Induction). Emergency anaesthesia and intubation.
Authors: Dr. Attila Erőss, Dr. László T. Hetzman, Dr. András Petróczy, Dr. Ákos Sóti, Dr. Péter Temesvári

Version 2 — 2013.11.20., 2015.03.25.

Title: RSI (Rapid Sequence Induction). Emergency anaesthesia and intubation.
Authors: Dr. Attila Erőss, Dr. László T. Hetzman, Dr. András Petróczy, Dr. Ákos Sóti, Dr. Péter Temesvári

Version 1 — 2011.05.13.

Title: RSI. Rapid Sequence Induction. Emergency anaesthesia and intubation.
Authors: Dr. Attila Erőss, Dr. László T. Hetzman, Dr. András Petróczy, Dr. Ákos Sóti, Dr. Péter Temesvári

---

Complete English translation of "RSI SOP V5" (Hungarian Air Ambulance Nonprofit Ltd. HEMS procedure, V5 — 2024.05.06.). Translated faithfully from the source with nothing omitted and nothing added. Abbreviations retained from the original: NP = nasopharyngeal airway, OP = oropharyngeal airway, NRBM = non-rebreather mask, MILS = manual in-line stabilization, BURP = Backward-Upward-Rightward Pressure, HELP = Head-Elevated Laryngoscopy Position, ApOx = apnoeic oxygenation, ETI = endotracheal intubation, PTX = pneumothorax, CICO = can't intubate, can't oxygenate, DR = doctor, PM = paramedic.
